# The Prognostic Role of CD8^+^ T Lymphocytes in Childhood Adrenocortical Carcinomas Compared to Ki-67, PD-1, PD-L1, and the Weiss Score

**DOI:** 10.3390/cancers11111730

**Published:** 2019-11-05

**Authors:** Ivy Zortéa S. Parise, Guilherme A. Parise, Lúcia Noronha, Mirvat Surakhy, Thiago Demetrius Woiski, Denise B. Silva, Tatiana EI-Jaick B. Costa, Maria Helena C. P. Del-Valle, Heloisa Komechen, Roberto Rosati, Melyssa Grignet Ribeiro, Marilza Leal Nascimento, José Antônio de Souza, Diancarlos P. Andrade, Mariana M. Paraizo, Marjorana Martini R. Galvão, José Renato S. Barbosa, Miriam Lacerda Barbosa, Gislaine C. Custódio, Mirna M. O. Figueiredo, Ana Luiza M. R. Fabro, Gareth Bond, Marco Volante, Enzo Lalli, Bonald C. Figueiredo

**Affiliations:** 1 Pelé Pequeno Príncipe Research Institute, 1532 Silva Jardim, AV., Curitiba, PR 80250-200, Brazil; 2 Faculdades Pequeno Príncipe, 333 Iguaçu Av., Rebouças, Curitiba, PR 80230-902, Brazil; 3 Hospital Infantil Joana Gusmão, 152 Rui Barbosa St., Florianópolis, SC 88025-300, Brazil; 4 Centro de Genética Molecular e Pesquisa do Câncer em Crianças (CEGEMPAC), UFPR, 400 Agostinho Leão Jr. Av., Curitiba, PR 80030-110, Brazil; 5 Serviço de Anatomia Patológica, Hospital de Clínicas, Universidade Federal do Paraná, 181 General Carneiro, Alto da Glória, Curitiba, PR 80060-900, Brazil; 6 Departamento de Medicina, PUCPR, 1155 Imaculada Conceição St., Prado Velho, Curitiba, PR 80215-901, Brazil; 7 Oxford Ludwig Institute for Cancer Research, Nuffield Department of Clinical Medicine, University of Oxford, Old Road Campus Research Build, Roosevelt Dr, Oxford OX3 7DQ, UK; 8 Hospital Pequeno Príncipe, 1070 Desembargador Motta Av., Curitiba, Paraná 80250-060, Brazil; 9 Ciência Laboratório Médico Ltda-Hospital Infantil Joana de Gusmão, 152 Rui Barbosa St., Florianópolis, SC 88025-300, Brazil; 10 Department of Oncology, University of Turin, San Luigi Hospital, regione Gonzole 10, Orbassano, 10043 Turin, Italy; 11 Institut de Pharmacologie Moléculaire et Cellulaire CNRS, 660 route des Lucioles, Sophia Antipolis, 06560 Valbonne, France; 12 Departamento de Saúde Coletiva, Federal University of Paraná, 280 Padre Camargo, Alto da Glória, Curitiba, PR 80060-240, Brazil

**Keywords:** adrenocortical carcinoma, histopathology, children, HLA, immune escape

## Abstract

Adrenocortical carcinoma (ACC) is a rare disease among children. Our goal was to identify prognostic biomarkers in 48 primary ACCs from children (2.83 ± 2.3 y; mean age ± SD) by evaluating the tumor stage and outcome for an age of diagnosis before or after 3 years, and association with ACC cluster of differentiation 8 positive (CD8^+^) cytotoxic T lymphocytes (CD8^+^-CTL) and Ki-67 immunohistochemical expression (IHC). Programmed death 1(PD-1)/Programmed death-ligand 1 (PD-L1) immunohistochemistry (IHC) in ACC was analyzed in a second, partially overlapping cohort (*N* = 19) with a similar mean age. All patients and control children were carriers of the germline *TP53* R337H mutation. Survival without recurrence for less than 3 years and death unrelated to disease were excluded. Higher counts of CD8^+^-CTL were associated with patients diagnosed with ACC at a younger age and stage I, whereas a higher percentage of the Ki-67 labeling index (LI) and Weiss scores did not differentiate disease free survival (DFS) in children younger than 3 years old. No PD-1 staining was observed, whereas weakly PD-L1-positive immune cells were found in 4/19 (21%) of the ACC samples studied. A high CD8^+^-CTL count in ACC of surviving children is compelling evidence of an immune response against the disease. A better understanding of the options for enhancement of targets for CD8^+^ T cell recognition may provide insights for future pre-clinical studies.

## 1. Introduction

The germline *TP53* R337H mutation is associated with a cluster of pediatric adrenocortical carcinoma (ACC) that is reported in Southern [1] and Southeastern Brazil [2], and accounts for the highest global incidence of childhood ACC [3,4]. The most frequent clinical features of childhood ACC typically include virilizing syndrome (VS) with accelerated development of pubic hair, facial acne, phallus growth, voice change, facial hair, hirsutism, and muscle hypertrophy or growth acceleration in 84% of the reported cases. Approximately 29% of patients present cortisol overproduction, sometimes with signs and symptoms of Cushing´s syndrome (CS) [5]. In contrast, features of adult ACC include secretory syndromes (60%) that are either a mixture of CS and VS (35%), CS (30%), or VS (20%) alone, feminizing syndrome (10%), and aldosterone-secreting carcinomas (2%) [6]. Older children (without a clear cut-off age) commonly exhibit features observed more often in adult ACC patients than in young children, which is consistent with the hypothesis that the ACC phenotype found in very young children is probably embryonic in origin [7,8,9].

Since the 1990s, global cooperative groups, particularly through the International Pediatric Adrenocortical Tumors Registry (IPACTR) and the Children’s Oncology Group (COG) [10], proposed improved treatment approaches for pediatric ACC. The current treatment for pediatric ACC is the combination of mitotane, cisplatin, etoposide, and doxorubicin, previously tested for adult ACC [11], adapted by COG [10], and put into use by Pediatric Oncology Centers [12]. In the absence of other efficacious regimens, the COG protocol also focused on improving surgical procedures and minimizing chemotherapy and mitotane toxicities, with the aim to increase cure rates for stage 3 and 4 patients. However, this trial was compromised by an unexpected negative outcome of retroperitoneal lymph node dissection (RPLND) in stage 2 patients. This outcome provided insights into response adaptation and growth of the remaining circulating ACC cells after surgical trauma [13]. Facilitated growth of these circulating ACC cells could be driven by inflammatory factors and/or diminished antitumor immune response.

Knowledge of malignant transformation of the childhood adrenal cortex has advanced significantly [14,15], but reliable prognostic biomarkers that can distinguish early- and advanced-stage ACC remain scarce, and clinical versus pathological findings are often inconsistent [5]. ACC in children under 5 years of age has more benign symptoms compared to older children with the same histopathological characteristics [16,17,18]. Elevated expression of the Ki-67 proliferation marker has been associated with poor prognosis in both adults [19,20,21] and older children [22]. However, this association between Ki-67 expression and poor prognosis in pediatric ACC is not consistent [17]. In contrast, other authors did not recommend Ki-67 immunohistochemistry in clinical practice to differentiate benign from malignant adrenocortical tumors without establishing a cut-off value for Ki-67 expression [23,24]. Furthermore, to increase the power of the prognostic value of Ki-67 in adult ACC, the Helsinki Score was proposed and validated as a measurement of mitosis, necrosis, and Ki-67 immunostaining [25,26]. Other prognostic modifiers should probably be considered. For example, hypercortisolism without virilization is usually associated with worse prognosis [5,27].

The role played by the immune system in human cancers has been extensively studied. Elevated exogenous or endogenous corticosteroid levels may downregulate the immune system [28,29]. The expression of HLA class I and II molecules in antigen presenting cells (APC) in tumors are key steps in the presentation of tumor antigens to T-lymphocytes (cytotoxic T lymphocytes; CTL) to generate an effective antitumor immune response [30,31,32]. CTL are cluster of differentiation 8 positive (CD8^+^), whereas T-helper and T-regulatory cells (Treg) are cluster of differentiation 4 positive (CD4^+^). CD8 is a transmembrane glycoprotein co-receptor of T-cell receptor (TCR) [33]. Mature CTLs (activated CD8^+^ T cells) are required to eliminate tumor cells, but impairment in cancer immunity may inhibit receptor signaling and lead to tumors that can evade the immune system [32]. Cancer cells regularly harness CTL antigen 4 (CTLA-4 or CD152) and programmed cell death-1 (PD-1 or CD279) immune-checking molecules to escape detection and elimination by the immune system [34,35,36,37]. This escape is usually mediated by elevated binding of the PD-L1 antigen in tumors to PD-1 receptors in CTL [34]. The PD-1 receptor and its ligand PD-L1 are an important part of the immune checkpoint mechanism and are important mediators of tumor-induced immune suppression that have emerged as important biomarkers for screening cancers amenable to immunotherapy. It is believed that most PD-L1-expressing tumors may respond to PD-1/PD-L1 inhibitors [32,37]. Indeed, there is some evidence that PD-L2 is associated with decreased survival and decreased CD8^+^ tumor-infiltrating lymphocytes in patients with esophageal cancer and could be a viable treatment target for many cancers, because it has a greater affinity for the PD-1 receptor than PD-L1 [38,39]. Studies regarding PD-1 and PD-L1 expression in ACC patients are limited; Fay et al. [40] reported that 10% PD-L1 expression in ACC cell membrane had no relationship with clinico-pathologic parameters or survival. Conversely, Tierney et al. [41] reported that PD-L1-positive cells accounted for only 2.9% (1/34) of ACC, contrasting with 44% (15/34) for PD-L2. These findings are consistent with the modest results from a multicenter phase IB trial that used antibody targeting PD-L1 [42].

Some solid tumors are known to be infiltrated by CD8^+^ cells, suggesting that these tumors are able to attract these cells through the presentation of specific antigens [43]. These antigens are bound by class I major histocompatibility complex (MHC) molecules, which help activate CTL and kill cancer cells [44,45]. Thus, the degree of CD8^+^ CTL infiltrated in solid tumors was found to be inversely correlated with tumor size, staging, and poor prognosis in colorectal cancer [46], prostate cancer [47], stomach cancer [48,49], melanoma [50,51], lung cancer [52], ovarian cancer [53], endometrial carcinoma [51,54], and other tumor types. 

The present study was designed to evaluate Ki-67, CD8^+^ CTL, PD-1, and PD-L1 expression in ACC of young children by immunohistochemistry (IHC). The main goal was to assess whether their expression is associated with age at diagnosis, disease free survival (DFS), and outcome.

## 2. Results

Demographic, clinical, and histopathological data from a cohort of 48 children with ACC are summarized in Table 1. All deceased patients ultimately died of disease within the first 4 years after diagnosis because of disease progression and/or relapses. The patient cohort included 30 girls and 18 boys, ranging from 5 d to 9.75 y of age with a mean age at diagnosis of 2.83 ± 2.3 y (mean ± SD), which presented virilization (40/48, 83%), CS (11/48, 23%), and hypertension (4/48, 8%). All stage 3 patients described in Table 1 were diagnosed either with vena cava invasion (*N* = 2), or had local invasion associated with contiguous invasion to the kidney with rupture of the tumor (*N* = 2). Stage assessment and treatment were performed according to the COG protocol [10] except for stage 2 RPLND, which was not performed in the present study. Adrenocortical adenoma (ACA), ACC with undetermined histology, deaths unrelated to ACC, or living patients with less than 3 y DFS after the last operation were excluded from the present study. Postoperative therapy was similar between all patients and according to the COG guidelines [10]. The minimum follow-up time for all patients free of disease was 3 y.

**Figure 1:**
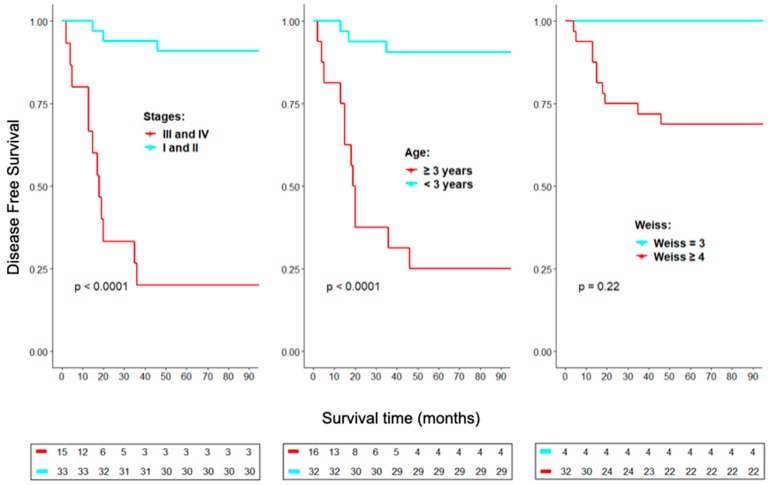
Kaplan-Meier analyses of staging, age at diagnosis, and Weiss score on disease free survival. All death cases were related to the disease. Surviving patients had more than 3 years without recurrence.

Advanced stage and age at ACC diagnosis ≥3 years were significantly associated with lower survival rates (Figure 1). Conversely, ACC with a Weiss score = 3 was not significantly associated with a higher DFS in comparison with cases with score 4 or higher.

### 2.1. Histology and Immunostaining of ACC Tumors

#### Ki-67, CD8 and PD-1/PD-L1 as markers for ACC prognosis

**Figure 2:**
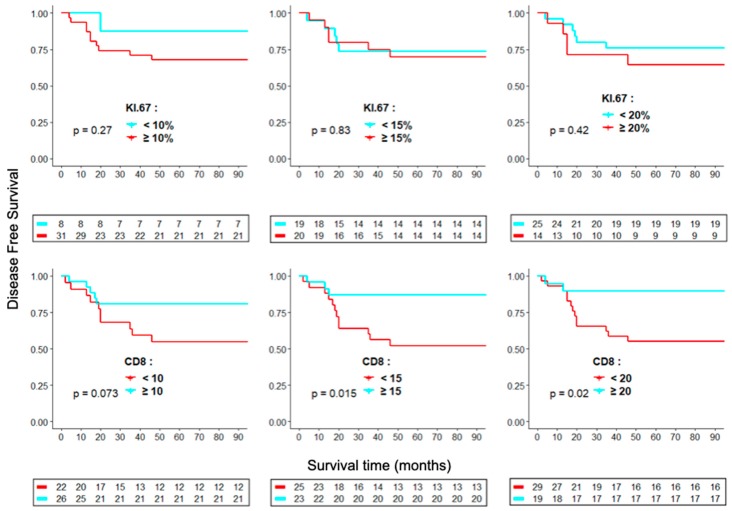
Kaplan-Meier analyses of overall survival for cluster of differentiation 8 (CD8) (cells/high power field (HPF)) and Ki-67 (% cells in HPF) indices at three cutoff levels. For each sample, ten high-power fields (HPF = 400×) with the most abundant ki-67 and CD8 distributions were selected and manually counted.

Evaluation of nuclear staining for Ki-67 in ACC cells was carried out by two independent observers (Thiago D. Woiski and Lúcia Noronha). We used previously reported counting methods for Ki-67 [21] and CD8 [46,55]. For each sample, ten high-power fields (HPF = 400×) with the most abundant distribution were selected and manually counted. Each tumor was scored at three mean cutoff levels (< or ≥10%, 15%, or 20% of 100 tumor cells) for the Ki-67 labeling index (LI), whereas mean absolute number of CD8 immunopositive cells in ten high-power fields (HPF = 400×) was expressed at three cutoff levels (< or ≥10, 15, or 20 cells per HPF). The relationship between either Ki-67 LI or CD8^+^ -CTL counts per HPF and overall survival was evaluated using Kaplan-Meier DFS analysis (Figure 2). We found significant prognostic values for two cutoffs of CD8^+^-CTL counts (15 and 20) but not for Ki-67 LI. Ki-67 also failed to reach significance in univariate (Table S1) and multivariate Cox analyses of stage as well as for ages above and below 3 y (Table 2).

**Figure 3:**
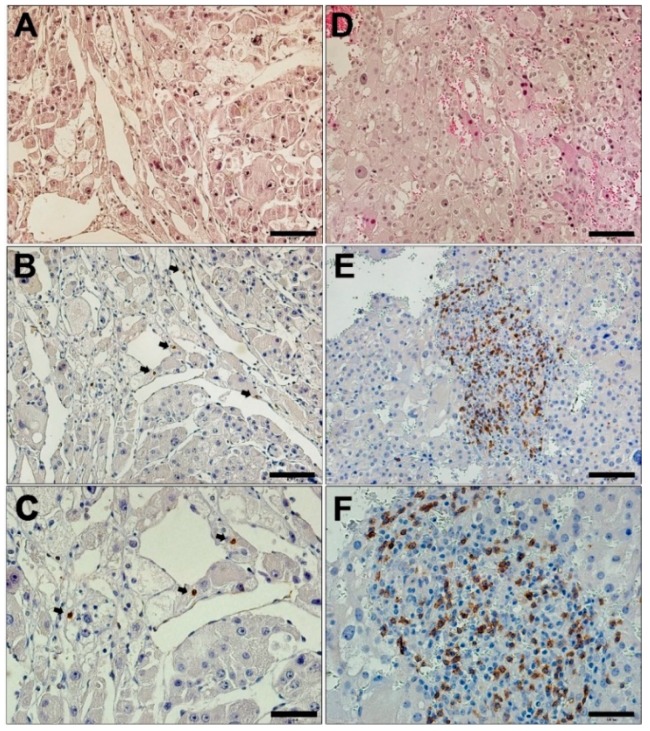
Immunohistochemistry of CD8 in adrenocortical carcinoma (ACC). (**A** and **D**) Low magnification (20×) images of hematoxylin and eosin stain (HE) of ACC. Diffuse, low CD8 immunostaining (**B**, 20×; **C**, 40×). Nodular CD8 immunostaining (**E**, 20×; **F**, 40×). Scale bar: A, D, B, and E = 100 µm; C and F = 50 µm.

Two different distribution patterns for CD8^+^-CTL were identified and termed nodular and diffuse. The multifocal distribution pattern was associated with higher counts of CD8^+^-CTL. In contrast, there was a diffuse pattern of CD8^+^-CTL associated with its lower counts (Figure 3).

**Figure 4:**
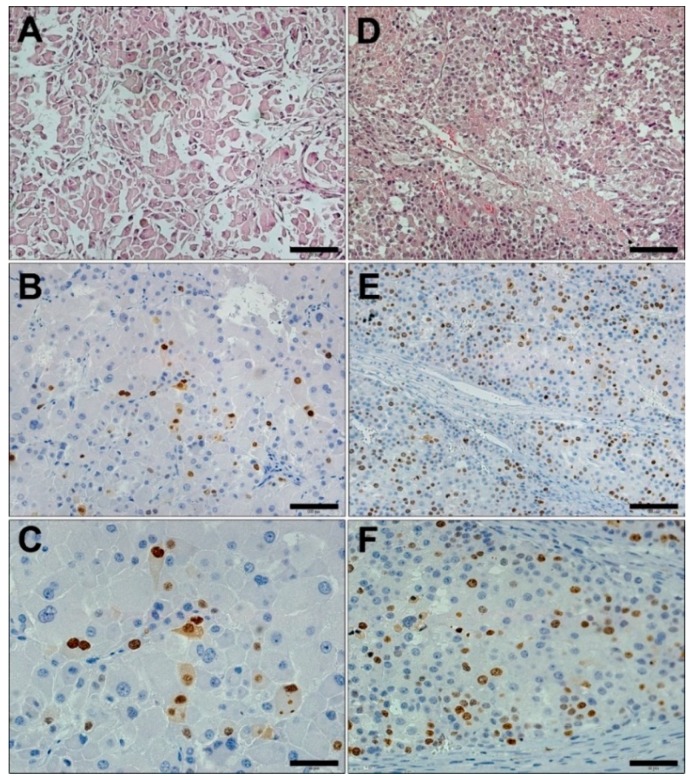
Ki-67 immunohistochemistry in ACC. Low magnification (20×) images shown in **A** and **D** depict HE staining of ACC. Low Ki-67 immunostaining is shown in **B** (20×) and **C** (40×). High Ki-67 expression is shown in **E** (20×) and **F** (40×). Scale bar: A, D, B, and E = 100 µm; C and F = 50 µm.

A single immunostaining pattern was observed for Ki-67-positive nuclei, which presented a wide range of counts between 1% and 70% (Figure 4).

Given the significant DFS difference between early stages (1 + 2) and advanced ACC (*p* < 0.00001; Figure 1), and the low number of ACC cases, additional analyses were performed to evaluate the prognostic value for Ki-67 LI and CD8^+^ positive CTL between two extreme prognostic groups. Group A included patients in stage 1 and 2 that were living disease-free for at least 3 y, and Group B was composed of all stage 3 and 4 patients that were alive or deceased, plus all stage 1 or 2 patients who died of disease. These two groups were compared using the Fisher’s Exact Test (Table 3) and analyses showed striking differences in CD8^+^-CTL counts and age of ACC diagnosis. In contrast, none of the Ki-67 LI values were statistically different between groups A and B.

Similarly, in a multivariate Cox analysis for advanced staging and age ≥3 y, or combining low CD8^+^-CTL counts and age ≥3 y, we were able to predict a significant correlation (Table 2).

**Figure 5:**
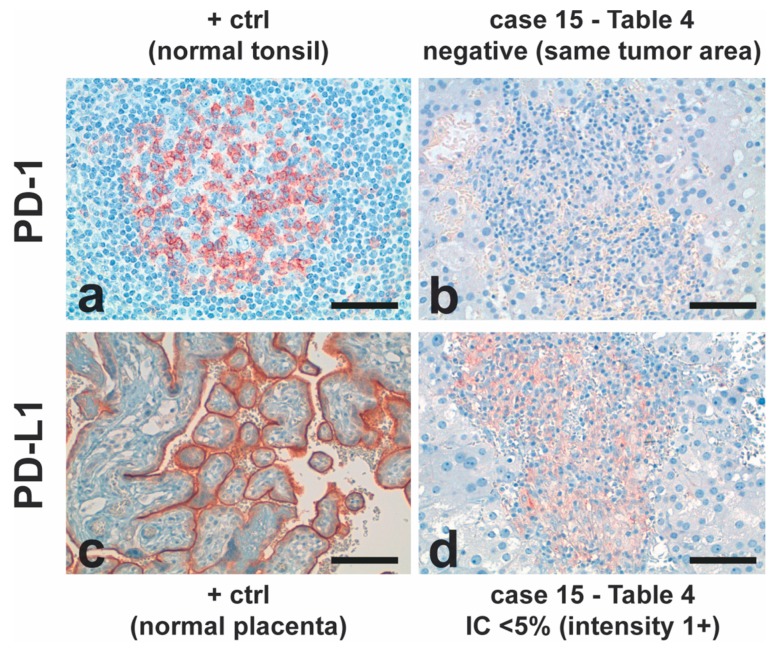
**Programmed death 1** (PD-1)/Programmed death-ligand 1 (PD-L1) staining profile in ACC and positive controls (+ ctrl). IC: immune cells. Scale bars: a, b, d: 50 µm; c: 100 µm.

Positivity in IC cells is usually graded into none, 1–5%, 5–10%, and >10%. The percentage was calculated as a percentage of immune cells that were positive (including lymphocytes and macrophages) from the total of immune cells. PD-L1-positive cells (<5%) were found in only four of the 19 tested ACC samples (21%), all of them exclusively in the tumor-associated immune cell component. PD-1-positive staining was not detected in immunoreactive tumor cells of any patient, only in positive controls (Figure 5).

Individual semiquantitative PD-1/PD-L1 results are presented in Table 4. The overall mean age of the children was 2.74 ± 2.4 y (mean ± SD). These findings suggest that the PD-1/PD-L1 pathway does not play a major role in ACC, and, given the modest results obtained for PD-1 and PD-L1, no further tests were performed to correlate those data to ACC prognosis.

## 3. Discussion

ACC assessment and treatment have evolved thanks to COG and other international consortia, but currently additional value is being placed on finding new prognostic biomarkers to predict outcome. To critically evaluate the markers in the present study, we considered different statistical models to evaluate prognosis and overall patient survival. We excluded adrenocortical adenomas (ACAs), undetermined adrenocortical tumors, deaths unrelated to ACC, wild-type germline *TP53*, and all cases of living patients with an uncertain outcome (<3 y of follow-up without recurrence or free of disease). Ki-67 expression in tumor cells was transformed into a categorical variable (< or ≥10%, 15%, or 20%), but failed to predict prognosis of young children with ACC carrying the *TP53 R337H* germline mutation. Kaplan-Meier/Log rank and univariate/multivariate Cox hazard analysis also did not support a predictive prognostic value for Ki-67 LI. Ki-67 expression also failed to differentiate stage 1 in living and disease-free patients from those with advanced tumors. Several attempts have been made to evaluate the prognostic significance of Ki-67 expression, and there is a consensus that Ki-67 is a reliable prognostic factor for adult ACC. A comparison among carcinoma groups was systematically performed according to European Network for the Study of Adrenal Tumors (ENSAT) staging [20,21,23,25,27,56]. However, the Ki-67 LI was not consistently associated with overall survival in stage 3 and 4 ACC [23].

Our patients are predominantly very young, with a mean age of 2.83 ± 2.3 y (± SD), who had typical clinical features represented by the predominance of virilization (83%) over CS (23%) and virilization combined with hypertension (8%). Children from the International Registry (IPACTR) who were over 13 y had a significantly lower survival rate than children under 3 y [5]. Interestingly, a pediatric ACC series from IPACTR with important differences in relation to our study (e.g., higher mean age, higher frequency of Cushing signs, lower frequency of virilization, absence of *TP53* germline mutations, inclusion of adenomas, and differences in disease-free survival) reported a significant association between high Ki-67 expression and worse prognosis [22]. However, these features are more similar to the adult ACC phenotype and the authors included adenomas and follow-up less than three years after treatment initiation, and did not describe the proportion of cases defined as stage 3 without vena cava invasion. These important methodological differences may affect the relevance of the Ki-67 LI associated with prognosis, and further studies with less heterogenous parameters are necessary to accurately evaluate Ki-67 LI use as a consistent disease marker.

Given the overall absence of PD-1 and PD-L1 immunoreactive tumor cells and 4/19 ACC tumor samples expressing PD-L1 (<5% (1+) in immune cells, it is less likely that the PD-1/PD-L1 immune escape pathway plays an important role in pediatric ACC. Similarly, only three (10.7%) PD-L1-positive cases were found in tumor samples among 28 adult ACCs [40]. Tierney et al. [41] found the low PD-L1 and strong PD-L2 expression in ACC tumors and stromal tissues than in ACAs, suggesting that PD-L2 could be a target for immunotherapy. Collectively, only a small number of patients could benefit from therapy using PD-1/PD-L1 inhibitors, which was recently demonstrated in a clinical trial [42].

ACC was reported as being a tumor type with the lowest immunologic response [57], which raises several questions about the feasibility of immunotherapy or therapeutic vaccine development against ACC. Fewer tumor infiltrating CD8^+^ T lymphocytes were reported to be associated with lower *HLA*-*DPA1* and *HLA*-*DPB1* expression in several pathologies [58], supporting the hypothesis that the loss of tumor immunosurveillance can have a devastating effect on tumor antigen presentation, loss of mature CTL, and patient outcome. Association between infiltrated CD8^+^ cells and a better ACC prognosis was also found in a multivariate Cox regression analysis for staging and patient age. Interestingly, *HLA*-*DPA1* is predominantly expressed in hematopoietic infiltrating cells in adrenocortical adenomas [37]. We observed that the multifocal (agglomerate) pattern of CD8^+^ cell infiltration predominates the diffuse distribution observed in ACC. The CD8^+^ focal pattern is more similar to the patterns reported in lung, breast, and colorectal cancers and melanoma than the diffuse pattern described in prostate or gastric cancers [59]. The multifocal pattern of CD8^+^ T lymphocyte distribution pattern in ACC could be influenced by the density of branching vessels (arborescent), with activated CD8^+^-cytotoxic T lymphocytes coming from the lymph nodes where antigen presenting cells may interact and activate immature cytotoxic T lymphocytes [32]. This pattern varies among tumors; for example in breast cancer, most of the tumor infiltrating CD8^+^ T lymphocytes are detected at the host–tumor interface than in the intra-tumor stroma [59].

## 4. Methods

### 4.1. Subjects and Samples

ACC tumor samples from 48 children were processed for immunohistochemistry (IHC). All ACC patients, three control children without cancer, and half of the parents were carriers of the germline *TP53* R337H mutation. Written informed consent was provided by the patients’ guardians. This study was approved by the Pequeno Príncipe Hospital Ethics Committee (Curitiba, Paraná state, Brazil, under ethic codes CAA: 0023.0.208.000-05 (2005), CAAE 0612.0.015.000-08 (2009 and 2012) and by the Hospital Joana Gusmão (Florianópolis, Santa Catarina state, Brazil, under ethic codes HIJG-006/2015, HIJG-003/2016, HiJG-006/2017) Ethics Committees for the IHC studies.

### 4.2. Immunohistochemistry 

Only primary adrenocortical carcinomas (ACC) carrying the germline *TP53* R337H mutation were included in IHC analysis. After deparaffinization and rehydration of 4-μm thick sections, endogenous peroxidase was inactivated with 0.5% Hydrogen Peroxide Block buffer (Sigma, Cambridge, MA, USA) for 10 min at room temperature. Antigen recovery was carried out by leaving the samples in Immuno Retriever buffer (Thermo Fisher™, Waltham, MA, USA) in a bath at 99 °C for 25 min. After cooling and washing, samples were incubated with primary antibodies overnight at 4 °C in a moist chamber. Tumor infiltrating T-cells and the Ki-67 labeling index were evaluated by IHC using the REVEAL-Polyvalent HRP Kit (Spring Bioscience, Pleasanton, CA, USA). The following primary antibodies were used: anti-CD8 (monoclonal rabbit, dilution 1:200, Thermo Fisher™) and anti-Ki-67 (monoclonal rabbit, dilution 1:200, Cell Marque™, Rocklin, CA, USA). All staining procedures included positive (e.g., tonsil) and negative controls (e.g., no primary antibody). The slides were then washed and incubated in Spring REVEAL complement buffer for 10 min at room temperature. Afterwards, the slides were washed and incubated with Spring REVEAL Conjugate for 15 min at room temperature. The slides were washed and revealed with DAB Chromogen (Thermo Fisher Scientific, Fremont, CA, USA) (1:1) and counterstained with Harris hematoxylin (Sigma-Aldrich, St. Louis, MO, USA).

The anti-CD8 and anti-Ki-67 immunostained slides were observed using a Zeiss^®^ AXIO SCOPE.A1 light microscope (Carl Zeiss Microscopy GmbH, Jena, Germany). For each sample, ten high-power fields (HPF = 400×) with the most abundant distribution were selected and manually counted. For Ki-67, the number of positive cells were counted for 100 cells per HPF and the percentage was used for statistical calculations as suggested [20]. Assessment of CD8^+^ T lymphocytes included counting the number of CD8^+^ membrane-stained tumor infiltrating lymphocytes in each HPF of the sample. The average was used for statistical calculations [46,55].

IHC for PD-L1 was performed in 18 ACC samples with the *TP53* R337H mutation according to a previous study [60]. Briefly, two commercially available antibodies for anti-PD-L1: antibody CAL10 (Biocare Medical, Pacheco, CA, USA) and the IVD versions of the PD-L1 IHC 22C3 pharmDx (DAKO Agilent Technologies, Santa Clara, CA, USA) were analyzed with the DAKO Autostainer Link 48 (Agilent Technologies, Santa Clara, CA, USA) and Ventana BanchMark (Ventana Medical System, Tucson, AZ, USA) platforms, respectively. Neoplastic cells were considered positive when any cell membrane staining (partial or complete) was present, and pure cytoplasmic immunoreactions were ignored.

IHC for PD-1 was performed as previously described [61] using the primary antibody EPR4877 (Abcam) at room temperature. Sections were further incubated with peroxidase-labeled secondary antibody for 30 min at room temperature. For antigen visualization, sections were immersed in 3-amino-9-ethylcarbazole plus substrate-chromogen for 30 min and counterstained with Gill’s hematoxylin.

The immunostained slides were observed on a Zeiss^®^ AXIO SCOPE.A1 optical microscope (Carl Zeiss Microscopy GmbH, Jena, Germany). For each sample, ten high-power fields (400×) were randomly selected and the average of the percentage of immunopositive cells was used for statistical calculations.

### 4.3. Statistical Analysis

We used R statistical software, version 3.5.0 R Core Team, 2018 [62] for all analyses. Survival curves were calculated according to the Kaplan-Meier method. The log-rank test was applied to detect significant survival differences between groups. The Kaplan-Meier non-parametric estimator was used to estimate the survival function of ACC patients for each of the covariates: staging, age group, Ki-67 LI percentages, CD8 counts (low or high), and a Weiss score ≥3 [63]. Univariable and multivariable Cox proportional hazard regression analysis was performed to identify the hazard ratios (HR) between pathological and clinical variables (staging, age group, Ki-67 group and CD8 group). The descriptive analysis was performed to verify the minimal quantities and percentages for the categorical variables (i.e. outcome, staging, age group, CD8 counts, and Ki-67 LI levels). Chi-square and Fisher’s exact tests were used to compare the dependency ratio between two categorical variables. The overall survival time was considered to be the elapsed time from the diagnosis until death or censorship (variable “time of follow-up”).

## 5. Conclusions

Relative risk prediction for childhood ACC beyond the classical clinical and histopathological parameters clearly depends on a number of parameters, including the status of the immune system, age at diagnosis, presence or absence of corticoid-mediated inhibition of the immune system, non-MHC genetic or epigenetic variants, and chromosome instability associated with p53 dysfunction, among others. The emerging evidence suggests that a better understanding of the CD8^+^ cytotoxic T lymphocytes could reveal variants in antigen presenting cells. However, restricted analysis to CD8^+^ cytotoxic T lymphocytes could lead to unexpected conclusions about ACC prognosis and outcome. Further studies and functional analyses are necessary to estimate HLA variants, the genotype versus phenotype of malignancy to identify the mechanisms of immune response in ACC. In addition, treatment should include attempts to modulate or improve immune response [64] at the primary tumor site because the overall survival for metastatic ACC is challenging and disappointing. These options may include enhancement of targets for CD8^+^ T cell recognition, as described for other human cancers [32,65,66] in combination with mitotane, cisplatin, etoposide, doxorubicin (EDPM), and/or targeted therapies.

## Figures and Tables

**Table 1:** Clinical and adrenocortical carcinomas (ACC) histological features.

Code	Gender	Age at Diagnosis	Clinical Manif.	Staging	Surgical Ressection	CT/M **	Recurrence	DFS	Outcome	Weight (g)	Weiss	CD8 Counts (Cells/HPF)	Ki-67 LI
1	F	1y10m	V	1	Total	No	No	3y1m	Well	77	8	17,8	10
2	F	1y6m	V	2	Total	No	No	8y7m	Well	127	5	22,7	7
3	F	3y4m	V	3	Total	CT/M	Yes	5m	DD	125	9	1,2	50
4	F	8y	V	3	Total	CT/M	No	10y3m	Well	300	3	22	14
5	M	2y4m	V+C	4	Total	CT/M	No	12y6m	Well	80	5	8,8	14
6	M	9m	NF	2	Total	M	No	6y	Well	300	6	18,2	15
7	F	8m	V+C	1	Total	No	No	3y	Well	62	7	3,2	53
8	F	2y4m	V	2	Total	M	No	3y3m	Well	275	5	4,2	18
9	M	1y1m	V	1	Total	No	No	6y9m	Well	12	5	37,8	37
10	F	1y9m	V+C	4	Total	CT/M	Yes	1y1m	DD	392	8	48,2	55
11	F	11m	V+C	1	Total	No	No	3y11m	Well	33	5	7,7	22
12	F	4y5m	V	3	Partial	CT/M	Yes	1y6m	DD	440	4	12,3	13
13	F	1y2m	V+C	2	Total	No	No	6y7m	Well	212.21	7	9,7	18
14	M	10m	V+C	2	Total	No	No	3y	Well	105	4	1,6	19
15	M	2y11m	V	2	Total	M	No	12y4m	Well	300	5	52,9	17
16	M	1y	ABM	2	Total	No	No	3y4m	Well	126	5	25,6	70
17	M	7y2m	No	2	Total	CT/M	No	4y10m	Well	238	6	24,9	35
18	F	4y5m	V	2	Total	CT/M	No	3y8m	Well	318	8	27,9	56
19	F	8m	V	1	Total	No	No	3y	Well	16	4	24,6	48
20	F	6y8m	V	4	Total	CT/M	Yes	1y3m	DD	342	9	16	70
21	F	10m	V+C	2	Total	No	No	11y1m	Well	135	3	3,2	2
22	F	1y8m	V+C	1	Total	No	No	6y8m	Well	*	*	25,3	11
23	M	9y9m	V+C	1	Total	CT/M	Yes	1y8m	DD	*	*	6	7
24	M	2y9m	V+H	4	Total	CT/M	Yes	2y11m	DD	625	7	0,7	15
25	M	8y5m	No	1	Total	CT/M	Yes	1y3m	DD	22	7	1,9	22
26	M	4y5m	V	1	Total	No	No	25y8m	Well	15	4	48,1	4
27	M	11m	ABM	2	Total	No	*	11y6m	Well	320	5	1	3
28	F	2y3m	V	1	Total	No	No	14y8m	Well	60	4	17,6	1
29	F	5y6m	V	2	Total	CT/M	Yes	3y10m	DD	690	8	1,9	20
30	F	5days	ABM	1	Total	No	No	15y1m	Well	10	3	39,7	10
31	F	6m	V	4	Total	CT/M	Yes	11/8m	Well	50	6	0,1	12
32	F	1y6m	V+H	1	Total	No	No	11y	Well	20	*	5,5	4
33	F	2y8m	V	1	Total	No	No	4y7m	Well	18	4	66,4	8
34	M	1y4m	V	2	Total	No	No	14y4m	Well	165	7	36,1	25
35	F	7y3m	V+H	4	Total	CT/M	No	4m	DD	400	4	34,9	10
36	F	5y3m	V	4	Total	CT/M	Yes	1y1m	DD	250	7	2,3	12
37	F	1y11m	V	1	Total	No	No	8y3m	Well	18	3	14,7	22
38	F	4y2m	V	4	Total	CT/M	Yes	1y7m	DD	750	6	4	12
39	F	8m	V	1	Total	No	No	5y8m	Well	100	6	4,4	12
40	F	2y7m	V	1	Total	No	No	21y	Well	20	*	22	*
41	F	7m	V+C	1	Total	M	Yes	15y3m	Well	100	*	68	*
42	M	2y7m	V	1	Total	No	*	21y	Well	50	*	43	*
43	M	1y10m	V	2	Total	No	*	4y9m	Well	170	*	0	*
44	F	1y3m	ABM	2	Total	No	No	11y9m	Well	265	*	76	*
45	M	3y4m	V	3	Total	CT/M	Yes	2m	DD	370	*	9	*
46	M	3y2m	V+C	4	Partial	CT	Yes	1y8m	DD	*	*	0	*
47	F	3y6m	ABM	4	Total	CT/M	Yes	3y	DD	123	*	0	*
48	M	2y10m	V+H	4	Total	CT/M	Yes	1y5m	DD	1050	*	10	*

* Not available; ** After total primary tumor resection, patients later developed metastases and were treated with CTM. DFS, Disease-free survival. F = female, M = male, V = virilization, C = Cushing, H = Hypertension, CT = chemotherapy, M = Mitotane, DD = died of disease; Y, yes, N, no; ABM, abdominal mass; Well means alive without signs of disease. CD8: cluster of differentiation 8, HPF: high power field, LI: labeling index.

**Table 2:** Multivariate Cox analysis of stage, age group, Ki-67 LI, and CD8^+^-cytotoxic T lymphocites (CTL) counts.

Parameters	Coefficient	Hazard Ratio	Standard Error	z	*p* Value
Stages 3 and 4	2.149	8.578	0.704	3.055	0.002
Age ≥ 3 years	1.746	5.729	0.698	2.5	0.012
Stages 3 and 4	2.658	14.26	0.86	3.091	0.002
Age ≥ 3 years	1.914	6.78	0.818	2.34	0.019
Ki-67 ≥ 20%	1.286	3.618	0.75	1.714	0.086
Age ≥ 3 years	2.647	14.11	0.787	3.362	<0.001
Ki-67 ≥ 15%	0.223	1.25	0.609	0.367	0.714
Age ≥ 3 years	2.635	13.94	0.798	3.303	<0.001
Ki-67 ≥ 20%	0.028	1.028	0.616	0.046	0.964
Age ≥ 3 years	2.552	12.84	0.659	3.872	<0.001
CD8 < 15 cells/HPF	1.377	3.963	0.657	2.096	0.036
Age ≥ 3 years	2.661	14.31	0.665	4.005	<0.001
CD8 < 20 cells/HPF	1.705	5.503	0.775	2.201	0.028

**Table 3:** Good (A) and worse (B) prognostic subgroups discriminated using different indices and parameters.

Parameters	Subroup A (Stages 1+2 Well) *n* (%)	Subgroup B (All Stages DD and 3+4 Alive) *n* (%)	*p*-Value
**Staging**			<0.001
1	16 (88.9)	2 (11.1)	
2	14 (93.3)	1 (6.7)	
3	0 (0)	4 (100)	
4	0 (0)	11 (100)	
**CD8^+^-CTL**			0.052
<10	10 (45.5)	12 (54.5)	
≥10	20 (76.9)	6 (23.1)	
-			0.008
<15	11 (44)	14 (56)	
≥15	19 (82.6)	4 (17.4)	
-			0.016
<20	14 (48.3)	15 (51.7)	
≥20	16 (84.2)	3 (15.8)	
**Ki-67**			0.218
<10%	7 (87.5)	1 (12.5)	
≥10%	18 (58.1)	13 (41.9)	
-			0.65
<15%	11 (57.9)	8 (42.1)	
≥15%	14 (70)	6 (30)	
-			1
<20%	16 (64)	9 (36)	
≥20%	9 (64.3)	5 (35.7)	
**Weiss_Score**			1
=3	3 (75)	1 (25)	
>3	20 (62.5)	12 (37.5)	
**Age**			<0.001
≥3 years	3 (18.8)	13 (81.2)	
<3 years	27 (84.4)	5 (15.6)	
**Outcome**			<0.001
DD	0 (0)	15 (100)	
Well	30 (90.9)	3 (9.1)	

DD means died of disease. CTL: cytotoxic T lymphocytes. The Chi-square and Fisher’s exact tests were used to compare the differences between subgroups.

**Table 4:** ACC stage and Programmed death 1 (PD-1)/Programmed death-ligand 1 (PD-L1) immunostaining.

Code	Age of Diagnosis	Clinical Manifestation	Stage	PD-L1 TC	PD-L1 IC	PD-1
1	1y10m	V	1	0	<5% (1+)	0
2	1y6m	V	1	0	0	0
3	3y4m	V	3	0	0	0
4	8y	V	3	0	0	0
5	2y4m	V+C	4	0	0	0
6	9m	NF	2	0	0	0
7	8m	V+C	1	0	0	0
8	2y4m	V	2	0	0	0
9	1y1m	V	1	0	0	0
10	1y9m	V+C	4	0	0	0
15	2y11m	V	2	0	<5% (1+)	0
16	1y	ABM	2	0	<5% (1+)	0
17	7y2m	No	2	0	<5% (1+)	0
19	8m	V	1	0	0	0
20	6y8m	V	4	0	0	0
26	4y5m	V	2	0	0	0
a	4y5m	V	3	0	0	0
b	1m22days	No	1	0	0	0
c	11m	V	2	0	0	0

V = virilization, C = Cushing, No = asymptomatic, ABM = abdominal mass. Codes 1 to 26 are as shown in Table 1. a, b, and c, other patients. TC: tumor cells, IC: immune cells.

## Supplementary Materials

The following are available online at https://www.mdpi.com/2072-6694/11/11/1730/s1, Table S1: Univariate Cox analysis of staging group, age group, Ki-67 LI, and CD8^+^-CTL counts.
