# Supplementary material for: The Prognostic Role of CD8+ T Lymphocytes in Childhood Adrenocortical Carcinomas Compared to Ki-67, PD-1, PD-L1, and the Weiss Score
Source: Cancers (Basel). 2019 Nov 5;11(11):1730. doi: 10.3390/cancers11111730 (PMC6896110; doi:10.3390/cancers11111730)
Supplement: Supplementary file 1 [file cancers-11-01730-s001.pdf]

Supplementary Material: Prognostic Role of CD8+ T Lymphocytes in Childhood Adrenocortical Carcinomas Compared to Ki-67, PD-1, PD-L1 and Weiss Score

Ivy Zortéa S. Parise, Guilherme A. Parise, Lúcia Noronha Mirvat Surakhy, Thiago Demetrius Woiski, Denise B. Silva, Tatiana EI- Jaick B. Costa, Maria Helena C.P. Del-Valle, Heloisa Komechen, Roberto Rosati, Melyssa Grignet Ribeiro, Marilza Leal Nascimento, José Antônio de Souza, Diancarlos P. Andrade, Mariana M. Paraizo, Marjorana Martini R. Galvão, José Renato S. Barbosa, Miriam Lacerda Barbosa, Gislaine C. Custódio, Mirna M. O. Figueiredo, Ana Luiza M R Fabro, Gareth Bond, Marco Volante, Enzo Lalli and Bonald C. Figueiredo

Table S1. Univariate Cox analysis of staging group, age group, Ki-67 LI, and CD8+-CTL counts.

Parameter	Coefficient	Hazard Ratio (95% CI)	z	*p* Value
Staging	2.8	17 (4.7–62)	19.0	0.000016
Age of diagnosis	2.6	13 (3.6–47)	16.0	0.000081
Ki-67 (15%)	0.13	1.1 (0.35–3.7)	0.05	0.82
Ki-67 (20%)	0.49	1.6 (0.5–5.4)	0.67	0.41
CD8 (15 cells/HPF)	−1.4	0.24 (0.067–0.84)	5.0	0.026
CD8 (20 cells/HPF)	−1.6	0.2 (0.046–0.9)	4.4	0.036

CD8: Cluster of Differentiation glycoprotein 8; LI:Labeling Index; CTL:Cytotoxic T Lymphocytes; CI: Confidence Interval; HPF: High Power Field (400×).

© 2019 by the authors. Licensee MDPI, Basel, Switzerland. This article is an open access article distributed under the terms and conditions of the Creative Commons Attribution (CC BY) license (http://creativecommons.org/licenses/by/4.0/).
